# Threonine Phosphorylation Fine-Tunes the Regulatory Activity of Histone-Like Nucleoid Structuring Protein in *Salmonella* Transcription

**DOI:** 10.3389/fmicb.2019.01515

**Published:** 2019-07-03

**Authors:** Lizhi Hu, Wei Kong, Dezhi Yang, Qiangqiang Han, Lin Guo, Yixin Shi

**Affiliations:** ^1^The State Key Laboratory of Virology, College of Life Sciences, Wuhan University, Wuhan, China; ^2^School of Life Sciences, Arizona State University, Tempe, AZ, United States; ^3^The Biodesign Institute, Arizona State University, Tempe, AZ, United States

**Keywords:** histone-like nucleoid structuring protein (H-NS), bacterial signal transduction, protein threonine phosphorylation, transcriptional regulation, post-translational modification

## Abstract

Histone-like nucleoid structuring protein (H-NS) in enterobacteria plays an important role in facilitating chromosome organization and functions as a crucial transcriptional regulator for global gene regulation. Here, we presented an observation that H-NS of *Salmonella enterica* serovar Typhimurium could undergo protein phosphorylation at threonine 13 residue (T13). Analysis of the H-NS wild-type protein and its T13E phosphomimetic substitute suggested that T13 phosphorylation lead to alterations of H-NS structure, thus reducing its dimerization to weaken its DNA binding affinity. Proteomic analysis revealed that H-NS phosphorylation exerts regulatory effects on a wide range of genetic loci including the PhoP/PhoQ-regulated genes. In this study, we investigated an effect of T13 phosphorylation of H-NS that rendered transcription upregulation of the PhoP/PhoQ-activated genes. A lower promoter binding of the T13 phosphorylated H-NS protein was correlated with a stronger interaction of the PhoP protein, i.e., a transcription activator and also a competitor of H-NS, to the PhoP/PhoQ-dependent promoters. Unlike depletion of H-NS which dramatically activated the PhoP/PhoQ-dependent transcription even in a PhoP/PhoQ-repressing condition, mimicking of H-NS phosphorylation caused a moderate upregulation. Wild-type H-NS protein produced heterogeneously could rescue the phenotype of *T13E* mutant and fully restored the PhoP/PhoQ-dependent transcription enhanced by T13 phosphorylation of H-NS to wild-type levels. Therefore, our findings uncover a strategy in *S.* typhimurium to fine-tune the regulatory activity of H-NS through specific protein phosphorylation and highlight a regulatory mechanism for the PhoP/PhoQ-dependent transcription via this post-translational modification.

## Introduction

Bacterial nucleoid-associated proteins play an essential role in chromosome organization. In enterobacteria, the H-NS is required for organizing and accommodating the chromosomal DNA (Gao et al., 2017). *Escherichia coli* H-NS is a 137 amino-acid protein that contains an N-terminal dimerization domain and a C-terminal DNA-binding domain separated by a flexible linker (Shindo et al., 1999). An amino acid sequence alignment analysis indicated that H-NS of *E. coli* and *Salmonella enterica* shared 95% identity with each other. It is known that the N-terminal 89 amino acids of H-NS comprise four α-helices, H1 (residues 2 to 7), H2 (residues 10 to 16), and H3 (residues 23 to 67) followed by a link sequence, and a fourth α-helix H4 (Esposito et al., 2002; Leonard et al., 2009; Arold et al., 2010). Two dimerization sites, located at H2 (site 1) and at H3 and H4 (site 2, between residues 57 and 83), interact in a manner of “head-to-head” and “tail-to-tail,” respectively, to create a chain of linked H-NS molecules and form a super-helical protein scaffold for DNA condensation (Arold et al., 2010). The DNA-binding region resides in the C-terminal 47-amino-acid fragment of H-NS protein that recognizes certain curved DNA (Shindo et al., 1995). Specifically, a short loop containing a so-called Q/RGR motif enables H-NS selectively to interact with the AT-rich DNA minor grooves (Gordon et al., 2011). This DNA binding feature also endows H-NS to be a global transcriptional regulator, which tends to silence transcription of virulence genes with high AT base contents in enteric pathogens (Porter and Dorman, 1994; Atlung and Ingmer, 1997; Lucchini et al., 2006; Banos et al., 2008; Zhao et al., 2008; Huttener et al., 2015; Prieto et al., 2016). In *Salmonella enterica*, H-NS plays a pivotal role in silencing horizontally acquired virulence genes that are grouped in clusters, referred to as *Salmonella* pathogenicity islands (SPIs), whose promoter sequences contain higher AT base contents than the resident genome (O’Byrne and Dorman, 1994; Kutsukake, 1997; Lucchini et al., 2006). On the other hand, H-NS-elicited repression is relieved by a wide range of transcriptional regulators that act on H-NS-bound promoters, thus removing this silencer or altering the H-NS-DNA structures. Among these regulators, the PhoP/PhoQ two-component system governs more than 40 genetic loci which are essential for *S*. Typhimurium virulence (Miller et al., 1989; Miller and Mekalanos, 1990). Many of these genes encode virulence factors conferring *Salmonella* resistance to host-secreted antimicrobial peptides (Fields et al., 1989; Gunn and Miller, 1996; Shi et al., 2004a), growth in Mg^2+^-depleted conditions (Soncini et al., 1996), survival in macrophages (Blanc-Potard and Groisman, 1997), and many other functions. The sensor PhoQ responds to environmental Mg^2+^, pH, and specific antimicrobial peptides (Garcia Vescovi et al., 1996; Bader et al., 2005; Prost et al., 2007; Prost and Miller, 2008), which in turn modifies the phosphorylated state of the DNA binding protein PhoP (for a review, see Groisman, 2001). Under the micromolar levels of Mg^2+^, the PhoQ protein phosphorylates the PhoP protein, resulting in expression of PhoP/PhoQ-activated genes, whereas under the millimolar levels of Mg^2+^, PhoQ mediates dephosphorylation of PhoP, which represses transcription of PhoP/PhoQ-activated genes (Castelli et al., 2000; Chamnongpol and Groisman, 2000). As a master regulatory system, the PhoP/PhoQ system also controls expression of various transcriptional regulators such as the two-component systems PmrA/PmrB (Kox et al., 2000) and RstA/RstB (Eguchi et al., 2004), as well as the MarR family member SlyA (Norte et al., 2003). According to our previous studies, transcription of many horizontally acquired genetic loci such as the *ugtL* and *pagC* genes are governed by a PhoP/PhoQ- and SlyA-dependent feedforward regulatory loop (Shi et al., 2004b; Zhao et al., 2008). Both *ugtL* and *pagC* genes are activated when the PhoP and SlyA regulators simultaneously bind to their promoter regions to release the H-NS protein. Recently, we demonstrated that another MarR-family member, EmrR, is a priming transcription activator that binds the *pagC* promoter prior to both PhoP and SlyA to displace H-NS, subsequently facilitates promoter binding of these successive regulators (Yang et al., 2019). Our previous study also showed that H-NS is essential for establishing the Mg^2+^-responsive transcriptional regulation of the PhoP regulon in *Salmonella* because deletion of the *hns* gene abolished the transcriptional repression of PhoP/PhoQ-activated genes in the millimolar levels of Mg^2+^ (Kong et al., 2008).

Protein phosphorylation has been widely studied in eukaryotes as a post-translational modification to play crucial roles in various biological processes such as enzymatic activity, protein-protein interactions, and cellular localization (Olsen et al., 2006). With the exception of well-elucidated histidine kinases from bacterial two-component regulatory systems (Goulian, 2010), characterization of protein phosphorylation in prokaryotes has seen limited progress probably due to lower phosphorylation level and tool shortage (Mijakovic and Macek, 2012). It took a long time to realize that eukaryotic-like Ser/Thr kinases also present in bacteria and a unique tyrosine kinase family in bacteria named BY-kinases also exist (Bakal and Davies, 2000; Grangeasse et al., 2007). The cross-talk between threonine phosphorylation and two-component signaling has also been reported (Lin et al., 2009). Post-translational modifications are identified in histone proteins of eukaryotes, which constitute a “histone code” recognized by effector proteins and thus play important roles in a variety of cellular processes (Jenuwein and Allis, 2001; Arnaudo and Garcia, 2013). It is shown that histone phosphorylation is important for chromatin remodeling linked to other nuclear processes (Rossetto et al., 2012). On the other hand, this post-translational modification on H-NS remains mostly to be investigated although a previous phosphotyrosine proteome study implied phosphorylation of H-NS at tyrosine residue 99 in *E. coli* (Hansen et al., 2013). A strategy to study phenotypic alternations stemmed from protein phosphorylation is to mimic its phosphorylated state *in vivo* by substituting phosphorylatable amino acid with a phosphomimetic analog. Indeed, the phenotypes of such a substitution had successfully revealed the consequence of many *in vivo* phosphorylation events (Mijakovic and Macek, 2012; Hansen et al., 2013; Horstmann et al., 2014).

From a total of 224 *Salmonella* phosphoproteins characterized from our quantitative phosphoproteome analysis (manuscript in preparation), 22 proteins were shared by *E. coli*, in which 79 phosphoproteins were identified in a previous study (Macek et al., 2008). In this study, we discuss a post-translational modification of H-NS protein, i.e., threonine phosphorylation in *Salmonella enterica* based on an observation from this phosphoproteome analysis. We show that protein phosphorylation at threonine 13 altered the H-NS structure dramatically and thus weakened its dimerization. A subsequent reduction in its DNA affinity caused transcription activation of the PhoP/PhoQ-dependent genes. Therefore, this study provides an example in which protein phosphorylation can modulate the function of H-NS through structural alternations.

## Materials and Methods

### Bacterial Strains and Growth Conditions

Strains used in this study were described in Table 1. All *Salmonella enterica* serovar Typhimurium strains were derived from the WT strain 14028s. Phage P22-mediated transductions in *Salmonella* were performed as described previously (Kwoh and Kemper, 1978). Bacteria were grown at 37°C in Luria-Bertani broth or in N minimal medium, pH 7.4, supplemented with 0.1% casamino acids and 38 mM glycerol (Snavely et al., 1991). MgCl_2_ was added to the required concentrations. When necessary, antibiotics were added at final concentrations of 50 μg/ml for ampicillin, 20 μg/ml for chloramphenicol, and 50 μg/ml for kanamycin. *Escherichia coli* DH5α was used as a host for the preparation of plasmid DNA.

**Table 1:** Bacterial strains and plasmids used in this study.

Strain or plasmid	Description	References or source
***S. enterica* serovar Typhimurium**		
14028s	wild-type	ATCC
YS17483	*hns-HA*::Cm^R^	Kong et al., 2008
YS17485	*hns-HA T13E*::Cm^R^	This work
YS17484	*hns-HA T13V*::Cm^R^	This work
YS17519	*hns-HA*::Cm^R^ *pagC-lacZ*::Km^R^	This work
YS17521	*hns-HA T13E*::Cm^R^ *pagC-lacZ*::Km^R^	This work
YS17520	*hns-HA T13V* ::Cm^R^ *pagC-lacZ*::Km^R^	This work
YS17529	*hns-HA*::Cm^R^ *pcgL-lacZ*::Km^R^	This work
YS17531	*hns-HA T13E*::Cm^R^ *pcgL-lacZ*::Km^R^	This work
YS17530	*hns-HA T13V*::Cm^R^ *pcgL-lacZ*::Km^R^	This work
YS17677	*hns-HA*::Cm^R^ *STM3595-lacZ*::Km^R^	This work
YS17679	*hns-HA T13E*::Cm^R^ *STM3595-lacZ*::Km^R^	This work
YS17678	*hns-HA T13V*::Cm^R^ *STM3595-lacZ*::Km^R^	This work
YS11591	*phoP-HA*	Song et al., 2008
YS18776	*phoP-HA T13E*::Cm^R^	This work
***E*. *coli***		
DH5α	F^-^ *sup*E44 Δ*lac*U169 (ϕ80 *lacZ*Δ*M15*) *hsdR*17 *recA*1 *endA*1 *gyrA*96 *thi*-1 *relA*1	Hanahan, 1983
**Plasmids**		
pKD3	rep_R6K_ γAp^R^ FRT Cm^R^ FRT	Datsenko and Wanner, 2000
pKD46	rep_pSC101_^ts^ Ap^R^ P*_araBAD_* γ β exo	Datsenko and Wanner, 2000
pCP20	rep_pSC101_^ts^ Ap^R^ Cm^R^ *cI*857 λP_R_	Datsenko and Wanner, 2000
pUHE21-2*lac*^q^	rep_pMB1_ Ap^R^ *lacI*^q^	Soncini et al., 1995
pUHE21-*hns-HA_WT_*	rep_pMB1_ Ap^R^ *lacI*^q^ *hns*-*HA_WT_*	Kong et al., 2008

### Construction of Chromosomal Mutations

The *hns-HA* strains harboring a substitution at the 13th residue (*T13E* mutant, YS17485; and *T13V* mutant, YS17484) were constructed by using a site-directed mutagenesis procedure described previously (Hu et al., 2016). Briefly, DNA fragments were PCR amplified using chromosomal DNA of *hns-HA*::Cm WT strain as template and primers 5′-T13E (5′-tacaatgagcgaagcacttaaaattctgaacaacatccgtgaacttcgtgcgcaggcaa-3′) and 3′-T13E (5′-tggggtcgtcagcggagaactcag-3′) for *T13E* mutant or 5′-T13V (5′-tacaatgagcgaagcacttaaaattctgaacaacatccgtgttcttcgtgcgcaggcaa-3′) and 3′-T13V (5′-tggggtcgtcagcggagaactcag-3′) for *T13V* mutant, and electroporated into a *Salmonella* WT strain containing plasmid pKD46 (Datsenko and Wanner, 2000). pKD46 was removed from transformants after bacterial cells were incubated at 37°C overnight. The substituted strains were selected as chloramphenicol resistant colonies and confirmed by DNA sequencing. All oligonucleotides were purchased from IDT (Integrated DNA Technologies).

### Characterization of Protein Phosphorylation Sites in H-NS Protein

**FIGURE 1:**
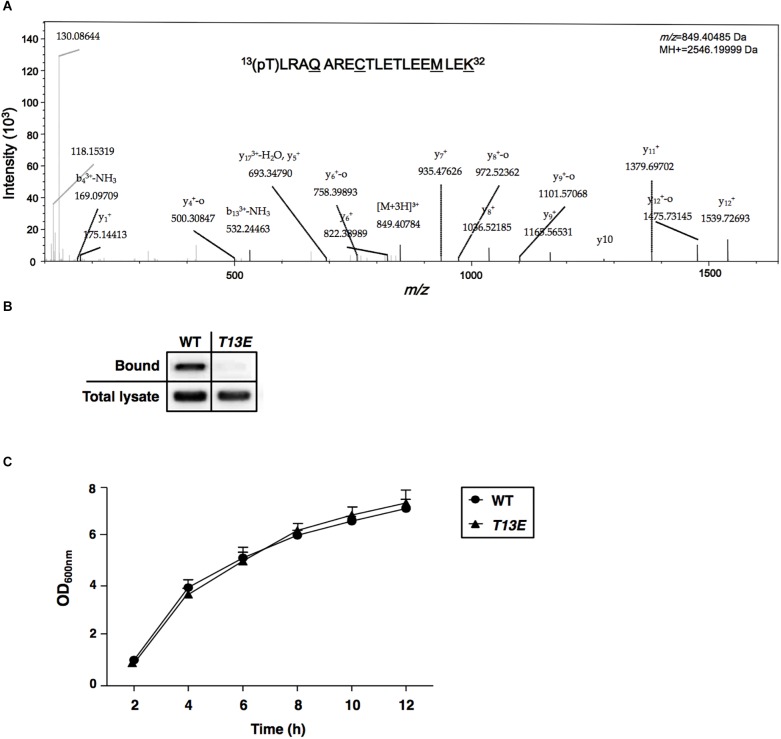
Characterization of T13 phosphorylation of H-NS in *Salmonella enterica* serovar Typhimurium. **(A)** The MS/MS spectrum of ^13^(pT)LRAQARECTLETLEEMLEK^32^. The phosphopeptide from *S.* Typhimurium wild-type strain (ATCC 14028s) was shown as an ion *m/z* 849.40485 with phosphorylated 13th threonine (pT). The underlined residues were modified from sample preparation: deamidated Q17, carbamidomethyl C21, oxidated M29, and dimethylated K32. Phosphorylation specific to T13, but not to T22 or T25, was specified by y_11_^+^ and y_12_^+^. **(B)** Immunoprecipitation assay of T13 phosphorylation of the H-NS_HA_-WT and H-NS_HA_-T13E proteins from the *hns-HA* wild-type strain (YS17483) and the isogenic *hns-HA T13E* mutant (YS17485), respectively. Bound represents the H-NS_HA_ protein that bound to the phosphothreonine-specific antibody. Total lysate represents the H-NS_HA_ protein present in the lysates before immunoprecipitation. The result was from one of the two independent experiments. Protein amount was quantified by ImageJ. **(C)** Growth of wild-type strain (YS17483) and *T13E* mutant (YS17485) in LB medium. Results were from two independent experiments. The OD_600nm_ value was measured after the original cultures were diluted with the medium used.

Data led to the identification of the H-NS T13 phosphorylation site (a relevant mass spectrum as shown in Figure 1A) were generated from a quantitative phosphoproteomics study (manuscript in preparation). *Salmonella* 14028s strain was cultured in 5 ml LB medium for 4 h, then collected by centrifuge at 6,500 rpm for 10 min and opened by sonication after suspended in 1 ml lysis buffer (50 mM Tris-HCl pH 7.5, 5 mM EDTA) supplemented with protease inhibitor cocktail (Sigma-Aldrich) and phosphatase inhibitor PhosStop (Roche Applied Science) by following manufacturer’s instruction. The total protein concentration of cell lysates or samples from other procedures carried out in this study was determined using the BCA Protein Assay Kit (Pierce). The total proteins (500 μg) were subjected to in-solution digestion with 10 μg trypsin (Promega), digested peptides were dimethyl labeled as suggested previously (Boersema et al., 2008) and then separated with a strong cation exchange (SCX) chromatography and eluted peptides were collected into eight fractions (Yu and Guo, 2011). Phosphopeptides were enriched from each SCX fraction (∼62.5 μg total peptides) using TiO_2_ micro-column by following a published method (Thingholm et al., 2006) and detected by a QE-HF mass spectrometer (Thermo Fischer Scientific) in a full MS/data-dependent MS^2^ (Top20) scan mode. All MS/MS data were analyzed using PD2.1 software. The Sequest search parameters were set as follows: parent ion tolerance 10 ppm and fragment ion mass tolerance 0.02 Da; carbamidomethyl cysteine as a fixed modification; serine, threonine, and tyrosine phosphorylation, methionine oxidation, and dimethyl (K)/(N-term)/2H(4)K/2H(4)N-term/13C(2)2H(6)K/13C(2)2H(6)N-term were variable modifications. The peptide charges were set to 2^+^ or 3^+^, allowing up to two missed cleavages.

### Quantitative Proteomic Analysis Using Stable-Isotope Dimethylation Labeling

The MS/MS method used in this study was dimethyl-labeling based relative quantification, not to get absolute abundances of the detected proteins (Ong and Mann, 2005). Bacterial cells grown in 10 ml LB for 4 h to an OD_600_ of 3.5–4.0 were harvested by centrifuge at 6,500 rpm for 10 min and then opened with sonication in 1 ml lysis buffer (50 mM Tris-HCl, pH 7.5, 5 mM EDTA) supplemented with a protease inhibitor cocktail (Roche Applied Science). Total proteins (500 μg) were precipitated with three volumes of a solution with 50% acetone, 49.9% ethanol, and 0.1% acetic acid. Pellets were resuspended in 100 μl resuspension buffer (8 M urea, 0.2 M Tris-HCl, pH 8.0, and 4 mM CaCl_2_). Proteins were reduced with DTT, alkylated with iodoacetamide and then digested with 10 μg trypsin (Promega). The stable isotope dimethyl labeling was conducted as described previously (Hu et al., 2016) with minor modifications. 4% formaldehyde (CH_2_O) and sodium cyanoborohydride (NaBH_3_CN) were added to samples (500 μg peptides in 250 μl 0.1 M NaAc, pH 5.9) for light labeling, 4% CD_2_O and NaBH_3_CN were added to samples for heavy labeling. The light-labeled and heavy-labeled samples were mixed at a 1:1 ratio and desalted with C_18_ Sep-Pak columns (Waters). All MS/MS analyses were performed on QE-HF mass spectrometer (Thermo Fischer Scientific) coupled with a nano-HPLC system (Tempo^TM^ ABSciex). All data were analyzed and quantified by using Proteome Discoverer Software Version 2.1 (PD2.1, Thermo Scientific). The Sequest search parameters were set as follows: parent ion tolerance 10 ppm and fragment ion mass tolerance 0.02 Da; carbamidomethyl cysteine as a fixed modification; methionine oxidation and dimethyl (K)/(N-term)/2H(4)K/2H(4)N-term were variable modifications. The peptide charge was set to 2^+^ or 3^+^, and up to two missed cleavages were allowed. To estimate the number of false positive protein identifications in a systematic fashion, the target-decoy approach was performed by searching both the target protein sequences and the reversed protein sequences in the database searching process (Elias and Gygi, 2007). The identification data were filtered to a 1% false discovery rate (FDR) using Percolator algorithm in Proteome Discoverer software platform (Brosch et al., 2009; Spivak et al., 2009). The proteins and peptides denoted with high FDR confidence (FDR < 1%) were exported and used for later analysis. The quantitative proteomic experiments were carried out in triplicates.

### Peptide Killing Assays

Antimicrobial peptide susceptibility assay was conducted by a modification of a previously described method (Shi et al., 2004a). Bacteria were grown in N minimal medium at pH 7.4, 10 mM MgCl_2_ for overnight at 37°C with aeration. Cultures were harvested and washed twice in N medium, pH 7.4. Cells were then diluted 1:100 into N medium, pH 7.4, 10 μM MgCl_2_ and incubated for 4 h at 37°C with aeration to an OD_600_ of 0.7–0.8 and diluted to 1–2 × 10^5^ per ml in N medium, pH 7.4, 10 μM MgCl_2_. Antimicrobial peptides were dissolved and serially diluted with either autoclaved distilled water (for magainin 2, Sigma-Aldrich) or 0.01% acetic acid (for human defensin HNP-1, BACHEM). 5 μl of the peptide solution was mixed with 45 μl of the bacterial culture in wells of a 96-well plate (Cell Culture Cluster, Costar^®^) to reach the final concentration. After incubation at 37°C for 90 min, mixtures were diluted 10 times with LB broth, and 50 μl of peptide-treated cells were plated on to LB agar plates and incubated at 37°C overnight. The number of colony-forming units (cfu) was counted and the percentage survival was calculated as follows: survival (%) = cfu of peptide-treated culture/cfu of no peptide culture × 100.

### Enrichment of the T13 Phosphorylated H-NS_HA_ Proteins

The *hns-HA* WT and *T13E* strains (YS17483 and YS17485) were grown in 25 ml LB at 37°C for 4 h to an OD_600_ of 3.6–3.9. Bacterial cells were spun down, washed twice with PBS, and resuspended in 1 ml lysate solution (137 mM NaCl, 2.7 mM KCl, 10 mM Na_2_HPO_4_, 1.8 mM KH_2_PO_4_, 1 mM PMSF phosphatase inhibitor PhosStop). The cells were opened by sonication and debris were removed by centrifuge at 10,000 rpm for 15 min. Bacterial lysates were adjusted to the same amount of total proteins, mixed with 1:500 anti-phospho-threonine antibody (Sigma-Aldrich) and then incubated at 4°C overnight. 20 μl EZview^TM^ Red Protein G Affinity Gel (Sigma-Aldrich) were added to the overnight mixture and gently mixed in an end-over-end mixer at 4°C for 4 h. Affinity gel beads were spun down at 1,000 rpm for 5 min and treated with one-bed volume of 50 mN NaOH. The elutes were collected and neutralized with 50 mN HCl immediately. Immunoblot analysis was carried out (see below) for detection of H-NS_HA_ proteins using an anti-HA antibody.

### Immunoblot Analysis

Bacterial cells cultured in the desired medium were collected by centrifuge and opened by sonication after resuspended in lysis buffer (50 mM Tris-HCl, pH 7.5, 5 mM EDTA). Bacterial lysates were normalized to the same amount of total proteins before being treated with the SDS-PAGE loading buffer. Proteins were separated in 12% SDS-PAGE and transferred to a nitrocellulose membrane (Bio-Rad) in a semi-dry electroblot cell (Bio-Rad). The HA-tagged and FLAG-tagged proteins were reacted with mouse monoclonal anti-HA and anti-FLAG antibodies, respectively (Sigma), detected with a horseradish peroxidase-conjugated anti-mouse antibody (Bio-Rad), and then visualized using ECL western blotting substrate (Pierce). A magnesium transporter protein CorA was used as the control and detected with a mouse multi-clonal antibody made in our laboratories. Relative protein amount was measured by ImageJ^1^.

### Chromatin Immunoprecipitation Assay (ChIP)

Bacterial cells cultured overnight were diluted 20 times in fresh N-minimal medium containing 0.01 mM Mg^2+^ for 4 h to an OD_600_ of 0.8–0.9. After normalized to the same OD, bacterial cells were mixed with formaldehyde to a final concentration of 1% to crosslink H-NS to genomic DNA. Then, the ChIP assays were performed by following the protocol described previously (Kong et al., 2008). Promoter DNA fragments from bacterial lysates (Input) and immunoprecipitated samples (IP) were quantified by qPCR in QuantStudio III (Applied Biosystems, Thermo Fisher) using reaction in PowerUp^TM^ SYBR^®^ Green Master Mix with chromatin DNA fragments and primers as follows. 675 (5′-acacatcgttatctgtgc-3′) and 676 (5′-tgttacacctcgcgagag-3′) for *pcgL*; 505 (5′-tggaacgtcattgac-3′) and 676 (5′-tttattcccgctccg-3′) for *pagC*; 2677 (5′-attccgggaagtaaacaag-3′) and 2678 (5′-cacaacgcgaaacaacatg-3′) for *STM3595*; and 2529 (5′-agcagtgcaaaatgccgaag-3′) and 2530 (5′-tccgaccacggtttgttc-3′) for *rpoD*. The *pcgL* promoter DNA fragments from bacterial lysates (Input) and immunoprecipitated samples (IP) were also detected by 25-cycle PCR reactions, which were separated on 1% agarose gel and visualized by ethidium bromide (Supplementary Figure S1).

### β-Galactosidase Assay

β-Galactosidase assays were carried out in triplicates and the activity was determined as described previously (Kong et al., 2008). Data correspond to at least three independent assays conducted in duplicates.

### H-NS Cross-Linking Assay

Bacterial cells cultured overnight were diluted 20 times in fresh N-minimal medium containing 0.01 mM Mg^2+^ and grown at 37°C for 4 h to an OD_600_ of 0.9. Then, the culture was divided into two parts: one was used for cross-linking and another as control. *In vivo* cross-linking was carried out by adding cross-linker formaldehyde to the bacterial culture up to a final concentration of 1% for 15 min. The reaction was stopped by adding glycine to a final concentration of 125 mM. Bacterial cells treated by cross-linker and control were both collected by centrifuge and washed with PBS twice, and then opened by sonication. The chromosomal DNA components associated with H-NS protein were removed by treating cell lysates with DNase I (Fermentas). The total protein concentration of all samples was determined using the BCA Protein Assay Kit (Pierce) and normalized to the same levels. The samples were treated with the SDS-PAGE loading buffer at 37°C for 30 min and H-NS monomer and cross-linked dimer were detected by immunoblot assay. Data correspond to three independent experiments and bands were quantified by ImageJ. The dimer percentage was calculated by (amount of cross-linked dimer)/(amount of total H-NS protein). The H-NS_HA_-T13E dimer in *hns-HA T13E* strain was significantly decreased (*p* = 0.0072).

### Partial Trypsin Digestion

Bacterial cells cultured as indicated above were washed with PBS twice, then suspended in trypsin digestion buffer (10 mM Tris-HCl, pH 7.6, 100 mM KAc, 10 mM MgAc_2_ and 1 mM DTT) and opened by sonication. The total protein concentration of cell lysates was determined using the BCA Protein Assay Kit (Pierce) and then normalized to the same levels. Lysates were mixed with trypsin (Sigma-Aldrich) to a final concentration of 30 μg/ml, and incubated at 25°C for 0, 10, 20, 30 min, respectively. Enzymatic digestion was stopped by boiling sample aliquots mixed with SDS-PAGE loading buffer and then remained H-NS-HA protein was analyzed by immunoblot. Data correspond to three independent assays and quantified using ImageJ software.

## Results and Discussion

### *Salmonella* H-NS Protein Can Be Phosphorylated at the 13th Threonine Residue

We carried out a quantitative phosphoproteome analysis of a *Salmonella enterica* serovar Typhimurium WT strain (14028s) and characterized a phosphorylated peptide from bacterial cells cultured to log phase in LB medium. This phosphopeptide displayed as an ion with *m*/*z* (*z* = +3) 849.40485 and was predicted to carry^13^(pT)LRAQARECTLETLEEMLEK^32^ sequence from H-NS protein (Figure 1A). Phosphorylation should take place at threonine 13 (referred to as T13, herein), but not at threonine 22 or threonine 25, because of the presence of two ions with *m*/*z* (*z* = +1) of y_11_^+^ (1379.6970) and y_12_^+^ (1539.72693), respectively (Figure 1A). This threonine phosphorylation (i.e., T13 phosphorylation) in H-NS was further confirmed by an immunoprecipitation and immunoblot analysis using an *hns-HA* strain constructed in our previous study (Kong et al., 2008), which produced an H-NS protein carrying a C-terminal HA-tag (named H-NS_HA_-WT). Firstly, a mouse phosphothreonine-specific antibody was used to react with overall phosphothreonine-containing proteins from bacterial lysates, and secondly, these protein-antibody complexes were pulled down by EZview^TM^ Red Protein G Affinity Beads (Sigma-Aldrich) which specifically bound to IgG. Then, enriched proteins were resolved in an SDS-PAGE, and the H-NS_HA_-WT protein was detected by an immunoblot analysis using anti-HA antibodies. Our result showed that H-NS_HA_-WT protein in this *hns-HA* WT strain was enriched by immunoprecipitation and monitored by an anti-HA antibody (marked as Bound, Figure 1B). To further verify whether it was the phosphorylated T13 residue of H-NS protein that interacted the phosphothreonine-specific antibody, we constructed an isogenic *hns-HA* mutant (or *T13E*) in which the T13 codon (^37^ACT^39^) of the *hns-HA* gene was substituted with a glutamate codon (GAA) and thus produced T13E substituted protein (referred to as H-NS_HA_-T13E) in that the 13th glutamate residue could not be phosphorylated. The T13E substitution did not affect bacterial growth since the optical density of *T13E* mutant was similar to that of WT strain during a 12-h incubation period in LB medium (Figure 1C). Also, the substitution had no effect on the expression of the *hns* gene in *S*. Typhimurium cells since the H-NS_HA_-WT level in WT strain was similar to the H-NS_HA_-T13E level in *T13E* mutant (shown as total lysate, Figure 1B) when bacterial cells were grown in LB medium for 4 h. Unlike the H-NS_HA_-WT protein, H-NS_HA_-T13E protein at the same level from *T13E* cell lysate could not be enriched by the phosphothreonine-specific antibody used for the immunoprecipitation (Figure 1B), thus confirming that H-NS protein could be specifically phosphorylated at the T13 residue. Together, these observations provided evidence that *Salmonella* H-NS protein could be post-translationally modified through phosphorylation at the 13th threonine residue.

### T13 Phosphorylation of H-NS Partially Releases Repression of the PhoP/PhoQ-Dependent Transcription

**FIGURE 2:**
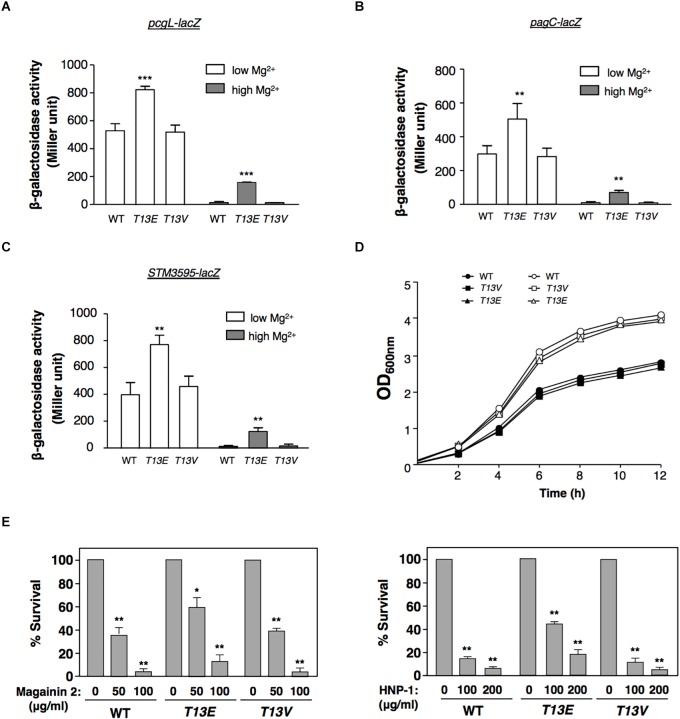
T13 phosphorylation partially releases the inhibitory effect of H-NS to the PhoP/PhoQ-activated genes. **(A)** β-Galactosidase activity from a *pcgL*-*lacZ* transcriptional fusion was determined in *S*. Typhimurium *hns-HA* wild-type strain (WT, YS17529), *T13E* mutant (YS17531), and *T13V* mutant (YS17530) grown in N medium with low (0.01 mM) and high (10 mM) Mg^2+^ for 4 h, respectively. ^∗∗∗^*P* < 0.001, vs. WT, *t*-test. **(B)** β-Galactosidase activity from a *pagC*-*lacZ* transcriptional fusion was determined in *S*. Typhimurium *hns-HA* wild-type strain (WT, YS17519), *T13E* mutant (YS17521), and *T13V* mutant (YS17520) grown in N medium with low and high Mg^2+^ for 4 h, respectively. ^∗∗^*P* < 0.01, vs. WT, *t*-test. **(C)** β-Galactosidase activity from an *STM3595*-*lacZ* transcriptional fusion was determined in *S*. Typhimurium *hns-HA* wild-type strain (WT, YS17677), *T13E* mutant (YS17679), and *T13V* mutant (YS17678) grown in N medium with low and high Mg^2+^ for 4 h, respectively. ^∗∗^*P* < 0.01, vs. WT, *t*-test. **(D)** Growth of wild-type strain (YS17483) and *T13E* mutant (YS17485) in N medium with low and high Mg^2+^, respectively. Results were from one of two independent experiments. The OD_600nm_ value was measured after the original cultures were diluted with the medium used. **(E)** Survival rates of *S*. Typhimurium wild-type strain (WT, YS17483), *T13E* mutant (YS17485), and *T13V* mutant (YS17484) after challenged by antimicrobial peptide magainin 2 (left panel) and human defensin HNP-1 (right panel) with indicated concentrations. Data correspond to three independent assays conducted in duplicate, and all values were mean ± standard deviation. ^∗∗^*P* < 0.01, ^∗^*P* < 0.05, vs. the strain untreated with tested peptide, *t*-test.

In physiological condition, glutamate residue was chemically similar to the phospho-threonine residue and had been successfully used as its phosphomimetic analog. Therefore, we used the *T13E* mutant to investigate whether T13 phosphorylation of H-NS exerted an effect on gene regulation on the premise that substitution of this threonine residue byglutamate in this mutant would make H-NS_HA_-T13E protein mimic the phosphorylated form of H-NS protein. A quantitative proteomic analysis, i.e., Stable-Isotope Dimethylation Labeling procedure (Hsu et al., 2003), was carried out to determine the levels of overall proteins labeled with ^1^H and ^2^H in the WT strain and *T13E* mutant, respectively, after bacterial cells were grown in LB medium for 4 h. Consistently, we found that levels of the PcgL and IraM proteins, which were encoded by two known PhoP/PhoQ-activated genes *pcgL* and *iraM* (Lejona et al., 2003; Battesti et al., 2012), were 2.9- and 3.2-fold higher in *T13E* mutant than those in WT strain, respectively (see the data *highlighted in yellow*, Supplementary Table S1). It was shown that H-NS served as a repressor for the transcription of the PhoP/PhoQ-activated genes (Kong et al., 2008). Based on these observations, we postulated that T13 phosphorylation of H-NS could partially release repression of the PhoP/PhoQ-activated transcription. It was worth noting that an *S.* Typhimurium *hns* null mutant constructed in a previous study carried an additional mutation in a two-component regulator gene *phoP* or sigma factor σ^S^ gene *rpoS* (Navarre et al., 2006). On the contrary, the expression level of the PhoP/PhoQ-activated genes in the *T13E* mutant was higher than that in the WT strain, indicating that the *phoP* locus in this mutant should be functional. This was confirmed by a DNA sequencing analysis using PCR fragments amplified from the *T13E* mutant which showed its *phoP* and *rpoS* coding regions remained identical to the WT sequences (data not shown). Actually, the proteomic analysis was carried out by using bacterial culture grown in LB medium containing an Mg^2+^ level around 0.3–0.4 mM (our unpublished data) which was higher than an Mg^2+^ level (0.01 mM) used to stimulate transcription of the PhoP/PhoQ-activated genes (Soncini et al., 1996). Thus, expression of most of the PhoP/PhoQ-activated genes was greatly reduced when bacterial cells were grown in LB medium, which could explain why very few proteins encoded by the PhoP/PhoQ-activated genes including those located in SPI-2 and SPI-3 were detected from our samples (Supplementary Table S1). We further verified the role of T13 phosphorylation in the PhoP/PhoQ-dependent transcription by constructing an *hns*-*HA* strain harboring a *lacZY* transcriptional fusion with the *pcgL* gene. In a PhoP/PhoQ-activated condition, i.e., N medium supplemented with 10 μM Mg^2+^ or referred to as low Mg^2+^ (Soncini et al., 1996), β-galactosidase activity in this *hns-HA pcgL-lacZY* strain was 1.6-fold lower than that in its isogenic *T13E* mutant (Figure 2A). In a PhoP/PhoQ-repressed condition, i.e., N medium supplemented with 10 mM Mg^2+^ or high Mg^2+^, *pcgL* transcription in WT strain was repressed to an undetectable level (Soncini et al., 1996). However, T13 phosphorylation appeared to partially release the *pcgL* transcription from this repression state under this condition because β-galactosidase activity in *T13E* mutant in high Mg^2+^ was raised to a level as high as ∼30% of the WT level in low Mg^2+^ (Figure 2A). However, this transcription activation of *pcgL* in *T13E* mutant was lower than that in an *hns* deletion mutant, which remained almost fully activated in high Mg^2+^ (Soncini et al., 1996; Kong et al., 2008). Similarly, transcription of other PhoP/PhoQ-activated genes (Miller et al., 1989; Soncini et al., 1996) including *pagC* and *STM3595* (a gene predicted to encode a phosphatase) was also upregulated by T13 phosphorylation of H-NS in both low and high Mg^2+^ (Figure 2B,C, and data not shown). In contrast with the *T13E* mutant, a T13 substitution mutant with valine residue (i.e., *T13V*) displayed a WT phenotype with regard to the PhoP/PhoQ-dependent transcription because β-galactosidase activity in *T13V* mutants harboring a *lacZY* fusion with the *pcgL*, *pagC*, and *STM3595* loci, respectively, was similar to their corresponding WT strains in low and high Mg^2+^ (Figure 2B,C). The H-NS_HA_-T13V protein in this substitution mutant, just like H-NS_HA_-T13E, did not react with the phosphothreonine-specific antibody (data not shown). Both *T13E* and *T13V* mutants grew similarly as the WT strain in both low and high Mg^2+^ in a 12-h growth period (Figure 2D), indicating that T13 substitutions had no effect on bacterial growth. The PhoP/PhoQ system plays an important role in the resistance of *S.* Typhimurium to a wide range of antimicrobial peptides (Fields et al., 1989; Gunn and Miller, 1996; Shi et al., 2004a). Consistently, upregulation of the PhoP/PhoQ-activated genes in the *T13E* mutant was correlated with an increased resistance to α-helical magainin 2 and β-sheet peptide human defensin HNP-1 because survival rate of the *T13E* mutant was 3.5- and 3.2-fold higher than that of the WT strain when bacterial cells were challenged by magainin 2 (100 μg/ml) and HNP-1 (200μg/ml), respectively (Figure 2E). Whereas the *T13V* mutant displayed similar phenotype to the WT strain regarding the resistance to these antimicrobial peptides. Therefore, we concluded that a new regulatory mechanism was established via T13 phosphorylation of H-NS to fine-tune the PhoP/PhoQ-dependent transcription. In addition to the PhoP/PhoQ-dependent genes, our quantitative proteomic analysis revealed that the T13 phosphorylation of H-NS should also be involved in the regulation of *Salmonella* pathogenicity island 1 (SPI-1) as well as several SPI-1 effectors, and methylation-dependent chemotaxis and flagellum biogenesis (seethe data shown in *red* and in *blue*, respectively, Supplementary Table S1). It remains to be investigated how T13 phosphorylation of H-NS contributes to these genetic loci.

### T13 Phosphorylation of H-NS Weakens Its Binding Affinity to the PhoP/PhoQ-Regulated Promoters

**FIGURE 3:**
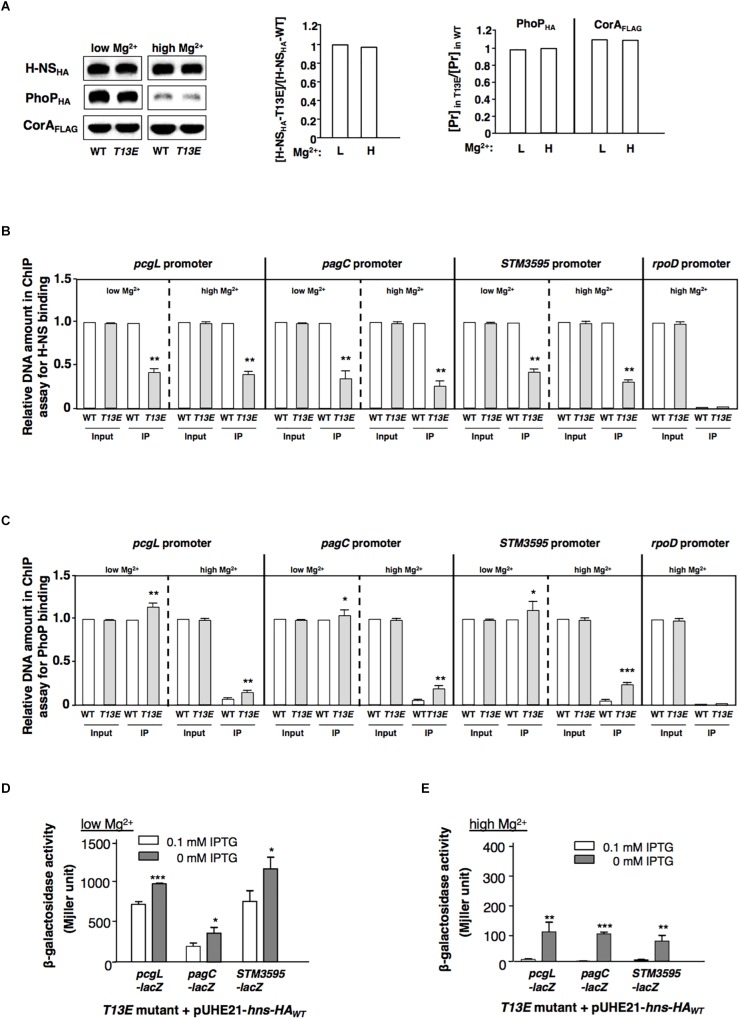
T13 phosphorylation lowers the affinity of H-NS to the PhoP/PhoQ-activated genes. **(A)** Immunoblot analysis of the total levels of H-NS_HA_ protein in the *hns-HA* strain (WT, YS17483) and the isogenic *T13E* mutant (YS17485) and the total level of PhoP_HA_ protein in the *phoP-HA* strain (WT, YS11591) and the isogenic *T13E* mutant (YS18776). Bacterial cells were grown in low and high Mg^2+^ for 4 h, respectively. Mg^2+^ transporter protein CorA was used as negative control. Protein amount was quantified by ImageJ and used to calculate ratios of H-NS_HA_-T13E vs. H-NS_HA_-WT with the formula: [H-NS_HA_-T13E]/[H-NS_HA_-WT] (middle panel); and ratios of the PhoP_HA_ and CorA_FLAG_ proteins (Pr) in wild-type strain (WT) and *T13E* mutant grown in low and high Mg^2+^ for 4 h, respectively, with the formula: [Pr]_highMg2+_/[Pr]_lowMg2+_ (right panel). **(B)** Chromatin immunoprecipitation-qRT-PCR assay for comparison of binding between H-NS_HA_-WT in the wild-type strain (WT, YS17483) and H-NS_HA_-T13E in *T13E* mutant (YS17485) to the *pcgL*, *pagC* and *STM3595* promoters in low and high Mg^2+^. The results were from three independent experiments and quantified by qRT-PCR (see section “Materials and Methods”). *Input* represents the total amount of target promoter DNA for the assay. *IP* presents the amount of target promoter binding to the H-NS_HA_ proteins. All WT results were taken to 1.0, respectively. ^∗∗^*P* < 0.01, vs. WT, *t*-test. **(C)** Chromatin immunoprecipitation-qRT-PCR assay for comparison of PhoP_HA_ protein to the *pcgL*, *pagC* and *STM3595* promoters between wild-type strain (WT, YS11591) and *T13E* mutant (YS18776) in low and high Mg^2+^. The results were from three independent experiments and quantified by qRT-PCR. *Input* represents the total amount of target promoter DNA for the assay. *IP* presents the amount of target promoter binding to the PhoP_HA_ protein. All Input results from wild-type strain (WT), and also the IP result from wild-type strain (WT) in low Mg^2+^ were taken to 1.0, respectively. ^∗∗∗^*P* < 0.001, ^∗∗^*P* < 0.01, ^∗^*P* < 0.05, vs. WT, *t*-test. **(D)** β-Galactosidase activity from *pcgL*-*lacZY*, *pagC*-*lacZY*, and *STM3595*-*lacZY* transcriptional fusions was determined in *T13E* mutants (YS17531, YS17521, and YS17679) harboring plasmid pUHE21-*hns-HA_WT_* grown in low Mg^2+^ with or without 0.1 mM IPTG. The results were from three independent experiments. ^∗∗∗^*P* < 0.001, ^∗^*P* < 0.05, vs. 0 mM IPTG, *t*-test. **(E)** β-Galactosidase activity from *pcgL*-*lacZY*, *pagC*-*lacZY*, and *STM3595*-*lacZY* transcriptional fusions was determined in *T13E* mutants (YS17531, YS17521, and YS17679) harboring plasmid pUHE21-*hns-HA_WT_* grown in high Mg^2+^ with or without 0.1 mM IPTG. The results were from three independent experiments. ^∗∗∗^*P* < 0.001, ^∗∗^*P* < 0.01, vs. 0 mM IPTG, *t*-test.

While T13 phosphorylation caused H-NS to reduce its regulatory activity, the protein level of H-NS_HA_-T13E in *T13E* mutant was similar to that of H-NS_HA_-WT in WT strain grown in both low and high Mg^2+^ conditions (Figure 3A). Therefore, we reasoned that T13 phosphorylation took place for weakening the binding affinity of H-NS to specific chromosomal regions including the PhoP/PhoQ-dependent promoters. A ChIP was conducted to compare the binding ability of H-NS_HA_-WT and H-NS_HA_-T13E to these promoters *in vivo*. We found that the affinity of H-NS_HA_-T13E to the *pcgL*, *pagC*, and *STM3595* promoters was reduced compared to that of H-NS_HA_-WT when bacterial cells were grown in low and high Mg^2+^ (Figure 3B). Concomitantly, a ChIP analysis using *phoP-HA* strains revealed that more PhoP_HA_ proteins in *T13E* mutant bound to the *pcgL*, *pagC*, and *STM3595* promoter regions under the same conditions (Figure 3C). This was in accordance with the competitive interaction of H-NS (the repressor) and PhoP (the activator) with the PhoP/PhoQ-dependent promoter regions (Kong et al., 2008). It also provided an explanation for the upregulation of these PhoP/PhoQ-dependent genes due to the T13 phosphorylation of H-NS. We found that in *T13E* mutant, H-NS_HA_-WT protein heterogeneously produced from plasmid pUHE21-*hns-HA* reduced the transcription of the *pcgL*, *pagC*, and *STM3595* genes to WT levels in both low Mg^2+^ and high Mg^2+^ (Figure 3D,E). We assumed that H-NS_HA_-WT protein could replace H-NS_HA_-T13E protein from the promoters of these genes, which subsequently restored their transcription to the WT levels. Consistent with the model for Mg^2+^-dependent autoregulation of the PhoP/PhoQ system (Garcia Vescovi et al., 1996), the PhoP_HA_ level in WT strain was significantly induced in low Mg^2+^ (Figure 3A). Although H-NS was shown to bind the *phoPQ* promoter (Kong et al., 2008), the PhoP_HA_ level in *T13E* mutant remained similar to that in the WT strain (Figure 3A). We postulate that H-NS phosphorylation at T13 should fine-tune transcription of the PhoP/PhoQ-regulated genes, but not be sufficient to modulate the expression of their regulator PhoP/PhoQ.

### The T13 Phosphorylation Modifies the Conformation of the H-NS Protein and Interferes Its Dimerization

**FIGURE 4:**
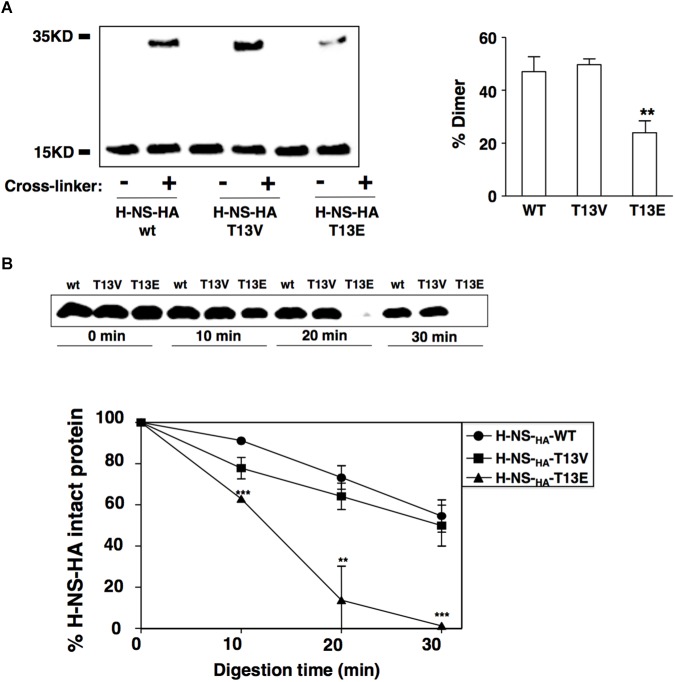
T13 phosphorylation reduces H-NS dimerization *in vivo* by inducing a conformational change. **(A)** *In vivo* cross-linking and immunoblot to monitor dimerization of H-NS_HA_ proteins in the wild-type strain (YS17483) and *T13E* mutant (YS17485). H-NS bands were quantified with ImageJ, and the percentage of H-NS dimer in each strain was calculated using the formula: Dimer/(Monomer + Dimer). **(B)** Partial trypsin digestion of H-NS_HA_ proteins. Crude extracts of the wild-type strain and *T13E* mutant were digested with trypsin (30 μg/ml) for 0, 10, 20, 30 min, respectively. Full-length H-NS_HA_ protein from each aliquot was analyzed and quantified by immunoblot. The results were from three independent experiments (^∗∗^*P* < 0.01; ^∗∗∗^*P* < 0.001).

It was demonstrated that two inter-monomeric salt bridge pairs were formed between the side chains of Arg14 and Glu38, and Arg40 and Glu26 from two dimerization domains at the first 46-residue N-terminal fragment of each H-NS protein (Bloch et al., 2003). We reasoned that the phosphoryl group added to the T13 might neutralize the positive charge of the adjacent Arg14, which subsequently weakened the electrostatic interaction between Arg14 and Glu38 and subsequently reduced dimerization of H-NS. To investigate this possible role of T13 phosphorylation in H-NS dimerization, we carried out an *in vivo* protein cross-linking assay with formaldehyde to determine the level of H-NS_HA_ dimers formed in bacterial cells. Consistent with the hypothesis, the protein level of the H-NS_HA_-T13E dimer in *T13E* mutant was ∼2-fold lower than that of the H-NS_HA_-WT dimer in WT strain or the H-NS_HA_-T13V dimer in *T13V* mutant (Figure 4A). To investigate whether reduced dimerization of the T13 phosphorylated H-NS subunits was caused by an internal rearrangement of specific amino acids residues including arginine and lysine, we conducted a partial trypsin digestion of H-NS_HA_-WT, H-NS_HA_-T13E and H-NS_HA_-T13V proteins on the premise that the conformational change would expose specific arginine and/or lysine residues in H-NS protein and subsequently alter its susceptibility to trypsin digestion. We found that the H-NS_HA_-T13E protein became extremely sensitive to the tryptic digest and was completely degraded after a 30-min treatment whereas 55.5% of the H-NS_HA_-WT protein and 50.2% of the H-NS_HA_-T13V protein remained intact (Figure 4B). We concluded that T13 phosphorylation should cause a dramatic change in H-NS conformation, allowing trypsin to access its target peptide bonds in H-NS T13E protein much easier than in H-NS_HA_-WT or H-NS_HA_-T13V protein.

## Conclusion

We have characterized threonine phosphorylation as a post-translational modification of the H-NS protein and found that it implements one means to modulate its accessibility to the specific chromosomal regions. The phosphorylation at T13 residue specifically causes structural alterations of H-NS without affecting its protein level. This modification actually decreases the H-NS dimerization and thus reduces the DNA binding ability probably because the T13 residue is a part of α-helices H2 forming the first dimerization site of H-NS protein (Arold et al., 2010). H-NS often forms polymers to bind large AT-rich DNA regions to silence transcription of horizontally acquired gene clusters and inhibit transcription of these regions (Lucchini et al., 2006). Absence of the H-NS protein has a strong impact on different horizontally acquired chromosomal regions (Navarre et al., 2006). Particularly, transcription of the PhoP/PhoQ-regulated genes was significantly activated in the absence of H-NS (Kong et al., 2008; Yoon et al., 2009). However, alterations in the H-NS structure through T13 phosphorylation are able to exert a moderate effect on, i.e., fine-tune, transcription of the PhoP/PhoQ-regulated genetic loci. Under the growth conditions used in this study, the *T13V* mutant, which produced an H-NS protein without phosphorylated 13th residue, did not significantly reduce the PhoP/PhoQ-dependent transcription compared to the WT strain. This implies that the T13 phosphorylation of H-NS protein occurs significantly when bacterial cells experience a specific condition. Our proteomic result showed that T13 phosphorylation downregulated the expression of SPI-1 and also methylation-dependent chemotaxis genes and flagellum biogenesis genes. At this time, it remains elusive with regard to the biochemical identity of the protein kinase(s) that mediates the T13 phosphorylation of H-NS.

## Author Contributions

LH, LG, and YS designed the experiments and wrote the main manuscript. LH, WK, DY, QH, LG, and YS carried out the experiments, collected the data, and analyzed the results. All authors reviewed the manuscript.

## Conflict of Interest Statement

The authors declare that the research was conducted in the absence of any commercial or financial relationships that could be construed as a potential conflict of interest.

## Footnotes

1 https://imagej.nih.gov/ij/

## Abbreviations

ChIP: chromatin immunoprecipitation assay
H-NS: histone-like nucleoid structuring protein
T13: threonine 13 residue of H-NS
T13E: glutamate substitution mutation of threonine 13 of H-NS
WT: wild-type.
