# Supplementary figures and images for: Threonine Phosphorylation Fine-Tunes the Regulatory Activity of Histone-Like Nucleoid Structuring Protein in Salmonella Transcription
Source: Front Microbiol. 2019 Jul 3;10:1515. doi: 10.3389/fmicb.2019.01515 (PMC6616471; doi:10.3389/fmicb.2019.01515)

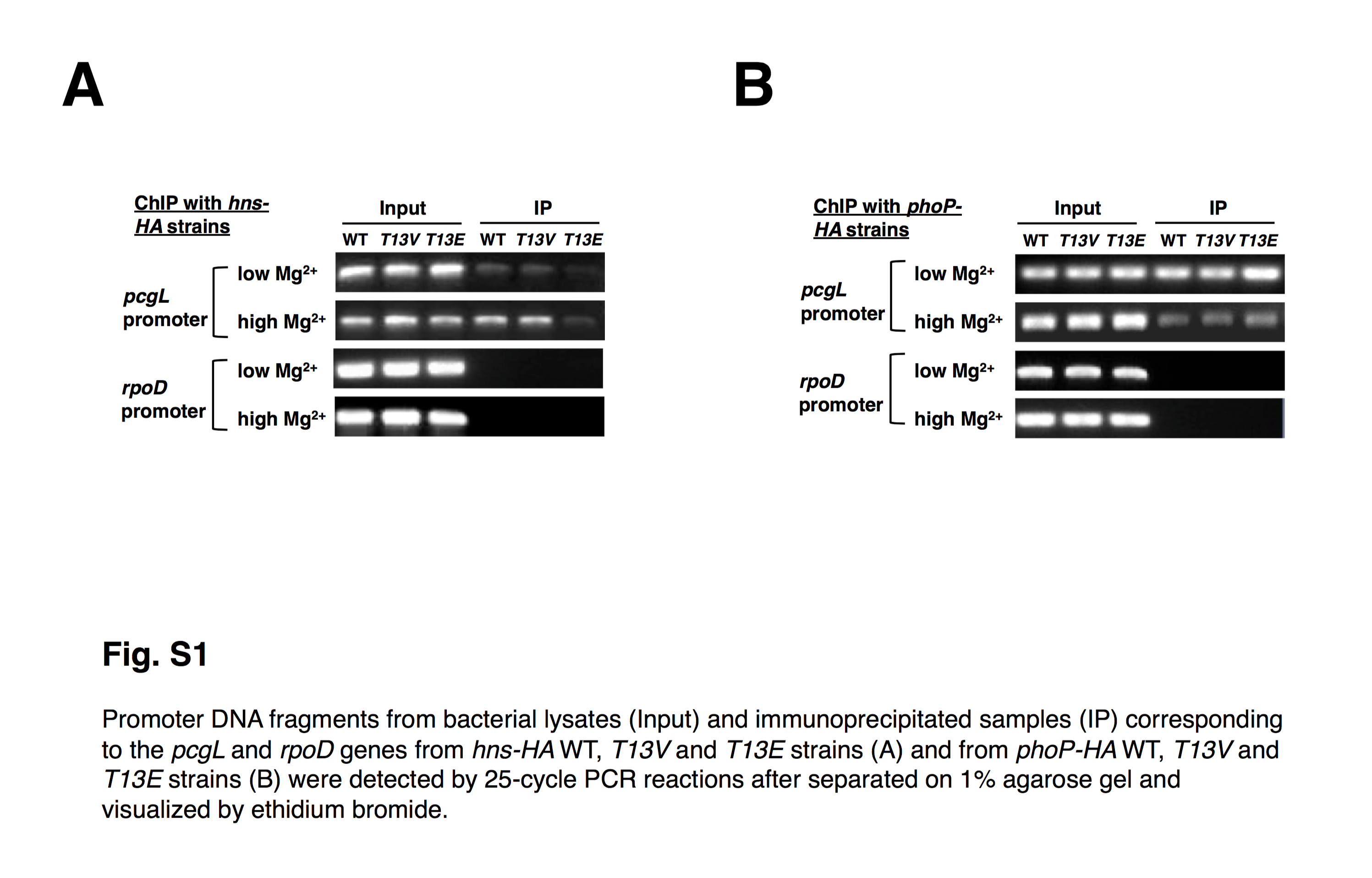

Supplement: Supplementary file 1 [file Image_1.TIFF]
